# The feasibility of point shear wave elastography (pSWE) in the assessment of pancreas stiffness in diabetic patients and healthy volunteers

**DOI:** 10.1371/journal.pone.0303098

**Published:** 2024-06-10

**Authors:** Fahad Farhan Almutairi

**Affiliations:** 1 Department of Radiologic Sciences, Faculty of Applied Medical Sciences, King Abdulaziz University, Jeddah, Saudi Arabia; 2 Animal House Unit, King Fahd Medical Research Centre, King Abdulaziz University, Jeddah, Saudi Arabia; 3 Smart Medical Imaging Research Group, King Abdulaziz University, Jeddah, Saudi Arabia; 4 Medical Imaging and Artificial Intelligence Research Unit, Center of Modern Mathematical Sciences and its Applications, King Abdulaziz University, Jeddah, Saudi Arabia; Universitatsklinikum Leipzig, GERMANY

## Abstract

Type 1 diabetes mellitus (T1DM) is an autoimmune disease characterized by the dysfunctional metabolism of carbohydrates, fats, and proteins caused by impaired insulin secretion and insulin resistance. This study investigated the feasibility of using point shear wave elastography (pSWE) of the pancreas by comparing the shear wave velocity (SWV) measurements of three anatomical areas in patients with T1DM and healthy volunteers. This study included 30 patients with T1DM (9 male, 21 female) and 23 healthy controls (11 men, 12 women). Two experienced certified operators performed the examinations and took the SWV measurements. The mean SWV of the entire pancreas parenchyma differed significantly between patients and controls (1.1 ± 0.29 and 0.74 ± 0.19 m/s, respectively; p ≤ 0.001). Moreover, the SWVs of the pancreatic segments were significantly different in patients and controls; the mean SWV values of the pancreas head, body, and tail (respectively) in patients vs. controls were 0.99 ± 0.36 vs. 0.76 ± 0.26 m/s (p  = 0.012), 1.1 ± 0.52 vs. 0.74 ± 0.23 (p ≤ 0.001), and 1.0 ± 0.34 vs. 0.73 ± 0.28 (p  ≤ 0.001). This study confirmed the feasibility of quantifying pancreas tissue stiffness with pSWE and revealed that patients with T1DM had higher pancreas tissue stiffness than controls. Further studies are required to determine the potential value of pSWE as a screening tool in patients with prediabetes.

## Introduction

Type 1 diabetes mellitus (T1DM) is an autoimmune disease characterized by the dysfunctional metabolism of carbohydrates, fats, and proteins caused by impaired insulin secretion and insulin resistance within the body. Over the decades, our understanding of the mechanisms involved in disease occurrence has substantially evolved. The main causal factor is the impaired production of insulin by the ß-cells of the pancreas, and insulin resistance is observed in the cells of the liver, skeletal muscles, and adipose tissues [1]. A recent systematic review and meta-analysis of 193 studies determined that the prevalence of T1DM was 9.5%, resulting in an extensive disease burden due to disease-related mortality and disability [2]. T1DM results in additional complications, such as cardiovascular complications and neuropathy [3]. Studies have highlighted that T1DM has a significant genetic component; thus, screening through imaging modalities may help reduce the burden of T1DM, especially for patients with a genetic risk of the disease, by allowing earlier diagnosis and intervention.

Recently, the application of ultrasound elastography to assess tissue stiffness has gained increasing attention. Studies have proposed that pancreas function can be evaluated with quantitative diagnostic modalities, such as ultrasound-based shear wave elastography (SWE) [4, 5]. SWE has been applied to assess tissue stiffness in the liver, breast, thyroid, and prostate [6–9]. SWE quantifies the velocity of shear waves and can thus be used as a non-invasive method to determine tissue elasticity. Studies have highlighted SWE’s potential clinical value in the early diagnosis and treatment of diseases [10, 11]. Hristov et al. investigated pancreatic cancer elasticity and revealed that ductal adenocarcinomas tend to be stiffer with a higher shear wave velocity (SWV) [12]. Moreover, increased tissue stiffness has also been reported in patients with chronic pancreatitis [13]. Studies have used SWE for pancreatic assessment in patients with pancreatic tumors and cancers [14, 15]. SWE has also been used to compare detection between patients with type 2 diabetes mellitus (T2DM) and healthy controls [16]. He et al. also studied the comparative detection in patients with T2DM and healthy controls using the shear wave method [17]. The results showed an increase in SWV in patients with T2DM, indicating an increased elasticity of pancreatic tissue. It is believed that the inflammatory process in the pancreas and the associated alteration in the pancreas tissue can be quantified using SWE technology; therefore, this technique could allow the early identification of prediabetes Although the use of non-invasive diagnostic imaging such as SWE would facilitate the early diagnosis of T1DM and the monitoring of the course of the disease, studies on SWE’s ability to detect T1DM are still limited. Therefore, this pilot study investigated the feasibility of pSWE of the pancreas in three different regions (the head, body, and tail) in patients with T1DM and healthy controls.

## Methodology

### Study design

**Fig 1:**
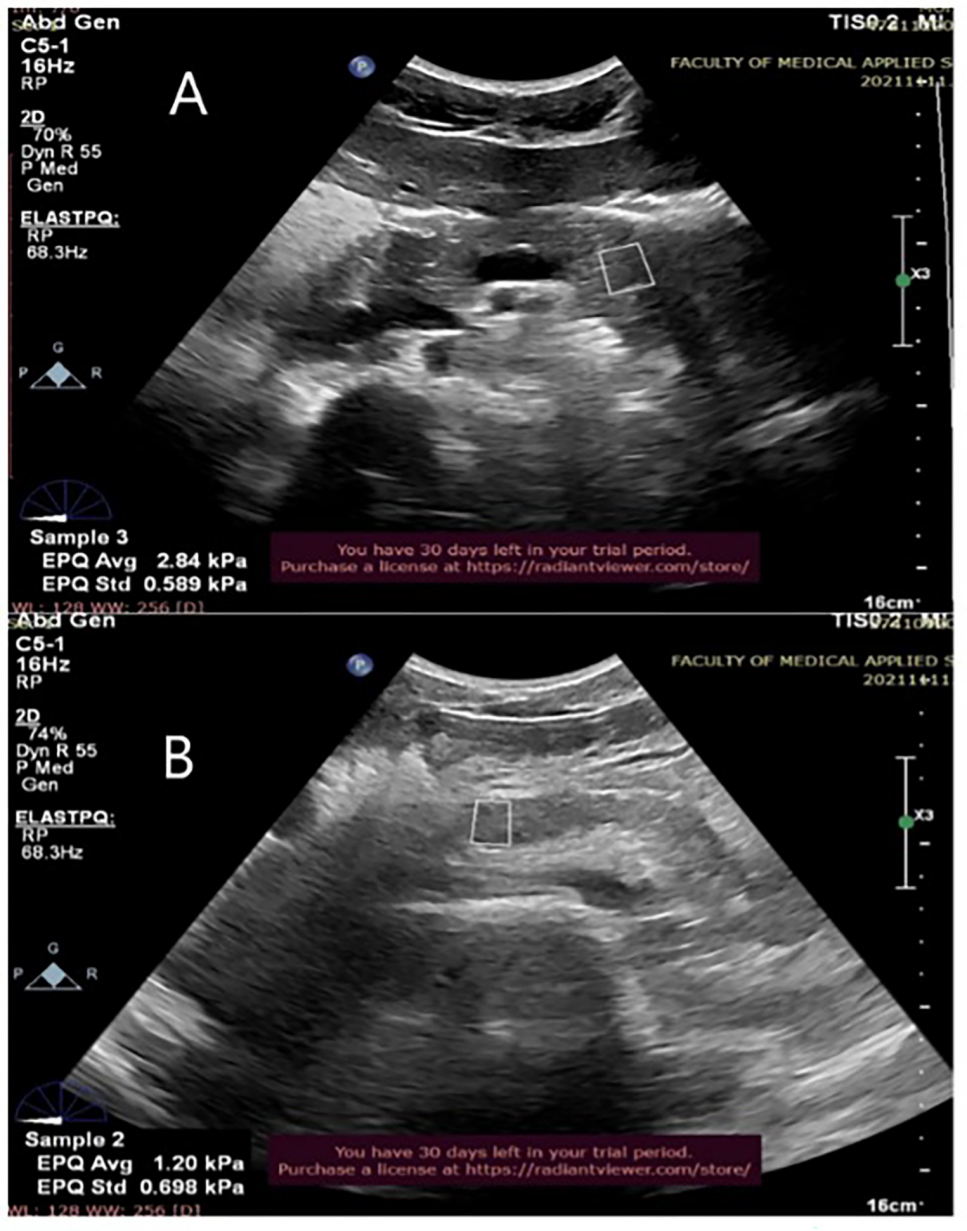
Shows an example of SWVs measurements in two different pancreas segments. A region of interest is positioned in the tail (A) and in the head of the pancreas (B).

This cross-sectional prospective study was approved by the local ethics committee (483–21). Ultrasound elastography examinations were performed by two certified clinical sonographers with extensive experience in elastography. Informed consent was distributed and signed by all participants. The individual pictured in Fig 1 has provided written informed consent (as outlined in PLOS consent form) to publish their image alongside the manuscript.

### Participants

This prospective study included of 30 patients with a previous diagnosis of T1DM and 23 healthy controls. Their baseline characteristics are summarized in Table 1. Patients and healthy participants were recruited and scanned at a diagnostic radiology department at King Abdul-Aziz University Hospital, Jeddah, Saudi Arabia.

**Table 1:** Study characteristics.

Characteristics	Patients (*n* = 30)	Control (n = 23)	p value
Gender, female: male	21:9	12:11	0.16
Age, years (SD)	58 (14)	25 (8)	< 0.001*
BMI, kg/m^2^ (SD)	28 (5)	22 (5)	0.18
Height, cm (SD)	160 (8)	164 (12)	0.18
Weight, kilograms (SD)	72 (16)	61 (15)	0.01*
Hypertension, Yes (%)	18 (60%)	0	< 0.001*
Smoking, Yes (%)	4 (13%)	4 (17%)	0.69
Family History, Yes (%)	27 (90%)	15 (65%)	0.03*

*SD* standard deviation, *BMI* body mass index, *Significant value, *p* < 0.05

### Ultrasound pSWE measurement

SWV measurements were obtained by an ultrasound scanner (Philips Elite Epic 7) with a 1–5 MHz curvilinear probe. The three anatomical segments of the pancreas (head, body, and tail) were examined and measured by ultrasound for each subject, and the SWV was measured in milliseconds. The pancreas was first identified in B-mode to start the scanning process. A general evaluation of the hepatobiliary system was then performed to rule out any disorders. The pancreas was measured longitudinally or from anterior to posterior, in the following dimensions: head, body, and tail. For every section, ten correct SWV measurements were acquired, and then mean was computed (Fig 1). The confluence of the superior mesenteric veins and spleen was measured in order to determine the border between the head and the body The anatomic structure was determined to be the tail. At the beginning of the scan, subjects were instructed to be in a supine position and take deep inspiration and hold until SWV measurements were taken. The region of interest (ROI) was accurately identified over each site, and the mean pancreas SWV was obtained and presented in m/s.

### Statistical analysis

Statistical analysis was performed with SPSS version 26 (SPSS Corporation, Chicago, IL, USA). Descriptive analysis and interquartile range (IQR)) were then computed. Pearson statistical analysis was used, and correlation coefficient was calculated. Normality test was performed using the Shapiro–Wilk test. Factors such as depth, age, weight, height, BMI, hypertension, smoking, and family history of diabetes were compared between study groups with Student’s t-test. A statistical p-value of <0.05 was considered statistically significant.

## Results

**Fig 2:**
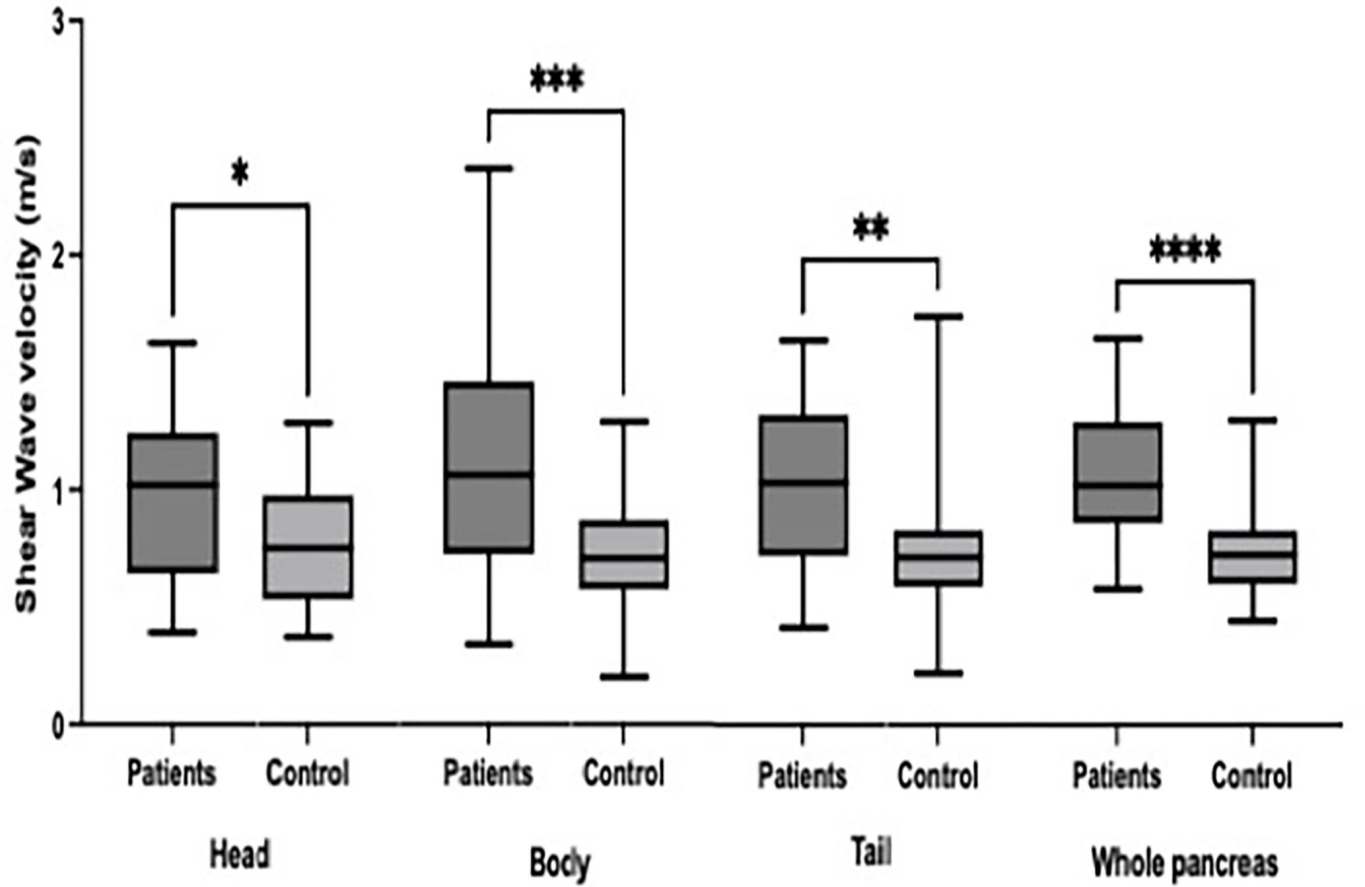
The mean SWV (m/s) for each pancreatic segment.

The study sample comprised 30 patients with diabetes (21 women and 9 men) and 23 healthy controls (12 women and 11 men). The demographic and clinical characteristics of the patients and controls are summarized and compared in Table 1. Significant differences (p < 0.0001) were noted for the following parameters: age, weight, hypertension, and family history of diabetes (Table 1). The mean SWVs of the entire pancreas parenchyma were 1.1 ± 0.29 m/s for patients with T1DM and 0.74 ± 0.19 m/s for controls (Fig 2). The mean age was 58 ± 14 years in the patient group and 25 ± 8 years in the control group. Patients and controls had a BMI of 28 ± 5 and 22 ± 5 kg/m^2^, respectively. A family history of diabetes mellitus was reported by 27 patients (90%) and 15 controls (65%); 13% of patients and 17% of controls were smokers (four participants in each group) (Table 1). The depth of the ROI, age, and height were positively correlated with the pancreas tissue in patients compared with controls (0.17, p = 0.02 vs. 0.07, p = 0.22; 0.20, p = 0.03 vs. 0.02, p = 0.41; 0.25, p ≤ 0.001 vs. 0.01, p = 0.61) (Table 2). The mean (SD) velocities of pancreatic segments in patients and controls were as follows: head, 0.99 ± 0.36 vs. 0.76 ± 0.26 m/s (p = 0.012); body, 1.1 ± 0.52 vs. 0.74 ± 0.23 (p ≤ 0.001); and tail, 1 ± 0.34 vs. 0.73 ± 0.28 (p ≤ 0.001) (Table 3). The SWVs of the three pancreatic segments (head, body, and tail) were significantly higher in the patient group; the mean (SD) SWV was 1.1 (0.29) m/s in patients and 0.74 (0.19) m/s in controls (p ≤ 0.001)

**Table 2:** Correlation of shear wave velocities (m/s) in both patients and control groups with depth, age, height, weight, and BMI.

	Stiffness (m/s)			
	Patients		control	
Factors vs stiffness	r	p	r	p
Depth (cm) vs pancreas	0.17	0.02	0.07	0.22
Age (years) vs pancreas tissue	0.20	0.03	0.02	0.41
Height (cm) vs pancreas tissue	0.25	< 0.00	0.01	0.61
Weight (kg) vs pancreas tissue	0.03	0.33	0.02	0.51
BMI (kg/m^2^) vs pancreas tissue	0.01	0.67	0.06	0.25

*BMI* body mass index

*P* values obtained by Spearman’s correlation test

**Table 3:** Shear wave elastography measurements in the three different pancreas segments (head, body and tail) of patients and control, SD Standard deviation, IQR Inter quartile range, level of significance P < 0.05.

	Stiffness (m/s)				
	Patients		control		p-value
Segment	mean (SD)	Median (IQR)	mean (SD)	Median (IQR)	
Head	0.99 (0.36)	1 (0.64–1.2)	0.76 (0.26)	0.75 (0.53–0.98)	0.012
Body	1.1 (0.52)	1.1 (0.73–1.5)	0.74 (0.23)	0.71(0.58–0.87)	0.001
Tail	1 (0.34)	1 (0.72–1.3)	0.73 (0.28)	0.72(0.59–0.83)	0.001
Mean	1.1 (0.29)	1(0.86–1.3)	0.74(0.19)	0.73(0.60–0.83)	<0.000

(Fig 2). The highest velocity was observed in the body of the pancreas in patients with diabetes (1.1 ± 0.52 m/s) and the lowest velocity was observed in the tail of the pancreas in the control group (0.73 ± 0.28 m/s) (Table 3).

## Discussion

This study assessed the feasibility of using pSWE to quantify pancreas stiffness in the three pancreas segments (head, body, and tail), compared the SWV of the pancreas in patients with diabetes with that of healthy individuals, and investigated the effects of various parameters on pSWE measurements Tables4–6.

**Table 4:** The multivariate analysis of the effect of having T1DM on pancreas head stiffness, while controlling for several variables (ANCOVA).

Source of Variation^1^		Type III Sum of Squares	df	Mean Square	F	Sig.	Coefficient of the Linear Equation
Corrected Model		1.580^a^	8	0.198	1.997	0.069	
Intercept		1.497	1	1.497	15.132	0.000	1.424
Group		0.917	1	0.917	9.267	0.004	0.727
Control variables:	Gender	0.096	1	0.096	0.971	0.330	0.115
	Age	0.333	1	0.333	3.363	0.073	-0.007
	BMI	0.068	1	0.068	0.691	0.410	0.009
	Hypertension	0.031	1	0.031	0.314	0.578	-0.071
	Family history diabetes	0.021	1	0.021	0.208	0.651	0.056
	Smoking	0.101	1	0.101	1.024	0.317	-0.151
	Pancreas dimension in head (cm)	0.157	1	0.157	1.591	0.214	-0.014
Error		4.451	45	0.099			
Total		48.614	54				
Corrected Total		6.031	53				

^1^ R Squared = .262 (Adjusted R Squared = .131)

**Table 5:** The multivariate analysis of the effect of having T1DM on pancreas body stiffness, while controlling for several variables (ANCOVA).

**Source of Variation** ^**1**^		**Type III Sum of Squares**	**df**	**Mean Square**	**F**	**Sig.**	**Coefficient of the Linear Equation**
Corrected Model		3.591^a^	8	0.449	2.532	0.023	
Intercept		0.430	1	0.430	2.424	0.127	0.758
Group		0.074	1	0.074	0.415	0.523	0.266
Control variables:	Gender	0.222	1	0.222	1.255	0.269	-0.178
	Age	0.016	1	0.016	0.092	0.763	0.002
	BMI	0.100	1	0.100	0.566	0.456	0.011
	Hypertension	0.128	1	0.128	0.721	0.400	-0.147
	Family history diabetes	0.093	1	0.093	0.522	0.474	0.118
	Smoking	0.158	1	0.158	0.893	0.350	-0.190
	Pancreas dimension in body (cm)	0.003	1	0.003	0.020	0.889	0.004
Error		7.977	45	0.177			
Total		62.432	54				
Corrected Total		11.568	53				

^1^ R Squared = .310 (Adjusted R Squared = .188)

**Table 6:** The multivariate analysis of the effect of having T1DM on pancreas tail stiffness, while controlling for several variables (ANCOVA).

Source of Variation^1^		Type III Sum of Squares	df	Mean Square	F	Sig.	Coefficient of the Linear Equation
Corrected Model		2.448^a^	8	0.306	3.492	0.003	
Intercept		0.784	1	0.784	8.947	0.004	1.453
Group		0.483	1	0.483	5.506	0.023	1.007
Control variables:	Gender	0.201	1	0.201	2.297	0.137	-0.169
	Age	4.953E-05	1	4.953E-05	0.001	0.981	8.609E-05
	BMI	0.284	1	0.284	3.239	0.079	0.018
	Hypertension	0.073	1	0.073	0.836	0.365	-0.110
	Family history diabetes	0.024	1	0.024	0.278	0.601	0.061
	Smoking	3.625E-05	1	3.625E-05	0.000	0.984	0.003
	Pancreas dimension in tail (cm)	0.355	1	0.355	4.053	0.050	-0.051
Error		3.944	45	0.088			
Total		50.566	54				
Corrected Total		6.393	53				

^1^ R Squared = .383 (Adjusted R Squared = .273)

The pancreatic segment elasticity and velocity measurements in patients were significantly higher than those in healthy controls. This finding is in line with the results of a study by Imamura et al., in which the pancreas stiffness in patients with diabetes was significantly higher than that of healthy controls (1.6 m/s vs. 1.1 m/s) [18]. However, their patient group was different than ours, which may explain why the difference between the SWVs of the two groups was higher in their study than in ours (0.5 m/s vs. 0.36 m/s). Studies have shown that liver fibrosis affects the stiffness of the abdominal organs, including the pancreas, kidneys, and spleen [19, 20]. However, a similar study conducted by Püttmann et al. comparing patients with T1DM and healthy individuals showed no significant difference; their study reported higher mean SWV values than ours (1.0, 1.2, and 1.1 vs. 0.99, 1.1, and 1.0 for the head, body, and tail, respectively) [21]. Although Püttmann et al. demonstrated the feasibility of using pSWE for the pancreas, their sample size may have been too small to provide reliable measurements [21].

Our study assessed the correlation between SWVs and demographic characteristics; the results revealed that pancreas stiffness in patients was positively correlated with depth, age, and height. Like our study, Püttmann et al. reported that stiffness was positively correlated with age across the pancreatic segments [21]. This study identified a positive correlation between depth and SWV measurements, which implies that the depth can affect the precision of SWV measurements. Phantom and clinical SWE studies investigating the effect of depth on elastography measurement have shown that depth may significantly impact the accuracy of the SWVs [22–24]. Therefore, our study showed that the depth of the pancreas was positively correlated with SWV measurements.

The SWVs of the head, body, and tail of the pancreas were significantly higher in the patient group compared with those in the control group. The highest velocity was observed in the body of the pancreas in patients, and the lowest velocity was observed in the tail of the pancreas in the control group. This finding is consistent with the results of studies by Yashima et al. and Mateen et al., in which the pancreas was stiffer in patients than in healthy controls [25, 26]. Contrary to our study, Püttmann et al. found that the head, body, and SWV measurements of the pancreas did not differ significantly between patients and controls [21]. However, despite the small sample size, the mean age of the patients and controls was quite similar.

Few studies have used SWE to quantify elasticity in different parts of the pancreas; these studies have reported normal SWVs, but discrepancies exist between SWV measurements. Stumpf et al. reported SWVs of 1.35, 1.41, and 1.20 m/s in the head, body, and tail, whereas Yashima et al. reported mean SWVs of 1.23, 1.30, and 1.20 m/s in the head, body, and tail, respectively [25, 27].

This study has several limitations. First, the association between imaging and laboratory biomarkers was not investigated. This association should be explored in a large-scale study. Second, we did not consider the duration of diabetes. Further studies should investigate the duration of the disease, as this can be an important factor in tissue stiffness quantification. Lastly, our study did not assess the correlation between the size and echogenicity of the pancreas and SWV measurements.

## Conclusion

Our study suggests that pSWE is a feasible method to assess pancreas stiffness and could be used as a screening tool for prediabetes. Patients with diabetes tend to have stiffer pancreas tissue than healthy controls. Further studies combining both pancreas stiffness and laboratory biomarkers are recommended.

## Supporting information

S1 File “Study_Raw_Data” is the raw data used in this study analysis. (XLSX)
